# Antibodies directed against bacterial antigens in sera of Polish patients with primary biliary cholangitis

**DOI:** 10.3389/fcimb.2024.1410282

**Published:** 2025-01-07

**Authors:** Alicja Bauer, Andrzej Habior

**Affiliations:** ^1^ Department of Biochemistry and Molecular Biology, Centre of Postgraduate Medical Education, Warsaw, Poland; ^2^ Clinic of Polish Gastroenterology Foundation, Warsaw, Poland

**Keywords:** PBC, bacterial infection, autoantibodies, molecular mimicry, autoimmunity

## Abstract

**Background:**

Primary biliary cholangitis (PBC) is a cholestatic, autoimmune liver disease with the presence of characteristic autoantibodies. The aim of the work was to determine the level of antibodies directed against bacterial antigens: *Chlamydia pneumoniae* (anti-*Cpn), Yersinia enterolitica (*anti*-Y.e*), *Helicobacter pylori* (anti-*Hp*), *Mycoplasma pneumoniae* (anti- *Mp.*) and *Escherichia coli* (*E.coli*) in sera of PBC patients. We also performed *in vitro* studies on the impact of the bacterial peptides on the specific antigen-antibody binding.

**Method:**

We screened 92 Polish PBC patients and sera samples from healthy donors and pathological controls. Autoantibodies and anti-bacterial antibodies were determined by commercially available ELISA kits. Specific inhibition of antibody binding was also detected by the in house ELISA method.

**Results:**

Anti-*Cpn*, anti-*Y. enterolitica*, anti-*Hp*, anti-*M. pneumoniae* and anti-*E. coli* antibodies were significantly more common in the group of PBC patients than in the pathological and healthy control groups: 74%, 40%, 84%, 39% and 69% respectively. The mean level of anti-*Cpn*, anti- *Y.e*, anti-*Hp and* anti- *M.p* in the PBC group was significantly higher than those in the healthy group (*p* < 0.001). and in patients with other liver diseases. In sera of patients with the presence of positive anti-mitochondrial antibodies (AMA), specific for PBC, anti-bacterial antibodies have been found in 80% vs. 50% in sera with AMA negative. We observed inhibition of specific antigen-antibody binding by the bacterial peptide: EClpP (*E. coli* caseinolytic protease) and adenine glycosylase from *E. coli* caseinolytic protease P, ClpP *Y.e* from peptide of *Y. enterolitica*, *Mp* PDC from *M. pneumonia* peptide and adenine glycosylase of *E. coli*. Bacterial factors influence the specific binding of antibodies to pyruvate dehydrogenase (PDC-E2), gp210 and KLHL12 (kelch-like peptide 12) antigens.

**Conclusion:**

Microbial mimics may be the major targets of cross-reactivity with human pyruvate dehydrogenase, gp210, and KLHL12 in PBC.

## Introduction

Primary biliary cholangitis (PBC) is a cholestatic, autoimmune liver disease, characterized by the existence of anti-mitochondrial (AMA) and antinuclear antibodies like anti-gp210 and anti-Sp100 in the patients’ sera (Ma et al., 2017; Bauer et al., 2021; Granito et al., 2021; You et al., 2022; Trivella et al., 2023; Wang et al., 2023; Xu et al., 2023; Wang et al., 2024). Antibodies to kelch-like peptide 12 (KLHL12) and to hexokinase 1 (HK1) have also been recognized as another PBC biomarker (Norman et al., 2015; Bauer et al., 2022). AMA is found in up to 90% of patients with PBC. AMA identifies a family of enzymes located in the inner membrane of mitochondria, coined the 2-oxo-acid dehydrogenase complexes (2-OADC), which primarily include PDC-E2, branched chain 2-oxo-acid dehydrogenase complex (BCOADC-E2), 2-oxo-glutaric acid dehydrogenase complex (OGDC-E2) and dihydrolipoamide dehydrogenase binding protein (E3BP) (Chen et al., 2016; Ergenc et al., 2023). The main subtype of AMA is anti-M2, directed against PDC-E2 (Juran and Lazaridis, 2008; Hitomi and Nakamura, 2023; Xu et al., 2023). The influence of the genetic background in the context of environmental factors, and interactions between effects in conferring predisposition to disease, has been evaluated. Specific disease-contributing alleles are not necessarily sufficient or necessary for the development of PBC, but they may be risk factors that modify the probability of the disease, through modulation of pathogenic processes (Donaldson et al., 2006). The described genetic factors connected with PBC, include the HLA locus and outside HLA loci (Invernizzi et al., 2008). The HLA class II locus on chromosome 6 includes groups of genes encoding HLA class II proteins that are strongly related with predisposition to various autoimmune diseases, also to PBC. In European populations, HLA-DQA1*04:01, HLA-DQB1*04:02, and HLA-DRB1*08:01 were described as influencing alleles, whereas HLA-DQB1*03:01 was recognized as a protective allele (Matsumoto et al., 2022; Tanaka et al., 2018)

Over the last 20 years, some attempts have been made to explain the relationship between environmental factors and the etiopathogenesis of the disease (Chen et al., 2013; Tanakaa et al., 2017; Tanaka et al., 2017; Jang et al., 2021; Wang et al., 2022). Many researchers, based on epidemiological data, suggest a higher incidence of autoimmune diseases during or after infection with certain microorganisms (Bogdanos et al., 2010). Bacterial infections have been studied as a major environmental factor in the etiology of PBC and one of the causes of loss of tolerance to mitochondrial autoantigens in PBC through the mechanism of molecular mimicry between human PDC-E2 and *E. coli* PDC-E2 (Dronamraju et al., 2010; Juran and Lazaridis, 2014; Leung et al., 2016; Lule et al., 2017; Tanaka et al., 2019). The cross-reactivity of AMA against some prokaryotic antigens has been described for several microbes, including *E. coli, Klebsiella pneumoniae, Proteus mirabilis, Staphylococcus aureus*, and *Salmonella Minnesota* (Bogdanos et al., 2010; Tanaka et al., 2019). Bogdanos et al. informed that IgG3 antibodies directed to the SxGDL[ILV]AE motif of *L. delbrueckii*-galactosidase were existing in AMA-positive PBC sera and cross-react with humanPDC-E2. *L. delbrueckii subspecies bulgaricus* is thus implicated as a probable environmental trigger for breaking tolerance against mitochondrial autoantigens. These antibodies also cross-reacted with human PDC-E2 (Bogdanos et al., 2005). Extensive research has confirmed the association of recurrent urinary tract infections (UTIs) with PBC (Smyk et al., 2011; Lule et al., 2017; Himoto et al., 2021). *Novosphingobium aromaticivorans*, a bacterium that metabolizes xenobiotics was also studied, suggesting that this type of bacteria can also associated with the etiology of PBC (Bogdanos et al., 2005).

Our study aimed to evaluate the level of antibodies directed against some bacterial antigens in sera of Polish patients with PBC. We determined anti-*Ch. pneumoniae*, anti-*Y. enterolitica*, anti-*H. pylori* anti-*M. pneumoniae* and *E. coli* antibodies. We verified AMA-positive and AMA-negative PBC patients’ sera for antibodies (Abs) against numerous infectious agents. We calculated also *in vitro* the influence of the bacterial peptide on specific binding antigen-antibody.

## Materials and methods

### Patients

We studied 92 Polish patients with PBC (87 women, 5 men; median age 51, range 26–74 years), diagnosed over the past 10 years. The diagnosis was based on the internationally accepted criteria (European Association for the Study of the Liver, 2017). The sera of patients positive for the hepatitis B surface antigen (HBsAg), anti-hepatitis A (IgM), and hepatitis C virus were excluded, and we also excluded patients with alcoholism, and AIH (autoimmune hepatitis)/PBC overlap syndrome. We also excluded patients with other autoimmune diseases. The control group contained serum samples from 30 healthy adult blood donors and pathological controls - 47 serum samples from patients with other autoimmune liver diseases: primary sclerosing cholangitis (PSC) and autoimmune hepatitis (AIH), 15 serum samples from patients with alcoholic liver cirrhosis (ALC). The study procedure was conducted according to the ethical guidelines of the Declaration of Helsinki and was accepted by the Centre of Postgraduate Medical Education’s ethical committee (Warsaw; approval number 71/PB/2019).

### Detection of autoantibodies and anti-bacterial antibodies

AMA and anti-nuclear antibodies were detected by commercially available kits (IMTEC-Human, Euroimmun; Germany and Inova Diagnostics; USA). Anti-*Cpn*, anti-*Y. enterolitica*, anti-*Hp*, anti- *M. pneumoniae*, and anti-*Chlamydia trachomatis* antibodies were tested by ELISA kits (Euroimmun; Germany), anti-*E.coli* antibodies were evaluated by ELISA kit (Chondrex, Inc, USA). Determination of antibodies was provided according to the manufacturer’s instructions

### Synthetic peptides

ECLpP - *E. coli* caseinolytic protease P, ClpP*Ye* – peptide of *Yersinia enterolitica*, *Mp* PDC – *mycoplasma pneumoniae* peptide and adenine glycosylase of *E. coli* were obtained from Yale University (New Haven, CT, USA).

**Table 1**. presents these synthetic peptides used in the study. The peptides corresponded to the region of similarity between bacterial peptide and human PDC-E2 or gp210, and to this region with residues from its vicinity. A negative control peptide was also included.

**Table 1:** The antigens used in this study.

Antigen	Amino acid sequence of peptides
Control peptide	LEHLKELIARNTWLTKKLPLSLSCF
Human PDC-E2	KKVGEKLSEGDLLAEIETDKATIGFEVQEEG
*E. coli* caseinolytic protease P	KASEGELLAQVEPED
ClpP *Y.e*	KATEGELLAQVEPED
*Mp* PDC	KKVGDTIKVDEALFVVETDKVTTELPSPYAG
Human Gp210	DRKASPPSGLWSPAYASH
*E.coli* adenine glycosylase	QRPPSGLWGGLY

### Detection of inhibition of antibody-antigen binding

1. Inhibition of antibody binding to human PDC-E2 by bacterial peptides; *ECLpP - E. coli caseinolytic protease P, ClpPYe* – peptide of *Yersinia enterolitica, Mp* PDC – *Mycoplasma pneumoniae* peptide
2. Inhibition of antibody binding to human gp210 antigen by bacterial peptides *E. coli* peptide - Adenine glycosylase of *E. coli* - QRPPSQLWGGLY, Gp210 antigen – DRKASPPSGLWSPAYASH.
3. Inhibition of antibody binding to human KLHL12 protein by bacterial peptide of *Yersinia enterolitica* - *Yersinia* adhesin A (YadA)
4. Inhibition of antibody binding to human HK-1 protein by bacterial peptide – caseinolytic protease subunit X of borrelia *Burgdorferi*

We detected the specific inhibition of antibody-antigen binding by the in-house ELISA method. To investigate whether the simultaneous reactivity to bacterial peptides was due to cross reactivity, competition ELISA was performed measuring antibody reactivity after exposure to these bacterial peptides and control peptide as liquid phase competitors (final concentrations: 0, 1, 2, 5, 9, 15, 30, 60, 150, 250 µg/mL).

All experiments were performed in 96-well microplates (maxi-sorp, Nunc, Denmark) coated with appropriate PBC-specific antigens. Plates (maxi-sorp, Nunc, Denmark) were coated overnight with suitable concentrations of the antigens (PDC-E2, Gp210, KLHL12, HK-1, respectively) in 0.1M bicarbonate buffer pH 9.6. After removing the antigen solution and rinsing the plate, non-specific absorption was prevented by the addition of 200 ml/well of 5% bovine serum albumin (BSA) in phosphate-buffered saline (PBS) and plates were incubated at 4°C overnight.

The bacterial peptides in consecutive dilutions were incubated for 2 h with shaking with PBC specific antibody at a constant dilution (1:100). After equilibrium had been reached, 100 ml of each solution was transferred to an ELISA plate coated with the PBC specific antigen, incubated for 1 h and washed. Next, the plates were incubated for 1 h at room temperature with peroxidase-conjugated anti-human rabbit IgG (Daco A/S Denmark, dilution 1:3000), and washed once again. The color reaction was developed by adding tetramethylbenzidine (TMB, Serva) for 15 min and stopping the reaction with 1N H_2_SO4. The optical density (OD) was measured at 450 nm. Each sample was tested in duplicate in at least three plates.

### Statistical analysis

Prevalence rates were compared between groups using the chi-square test and Fisher’s exact test. Continuous data were summarized as mean ± standard deviation (SD), and categorical data were summarized as frequencies. Continuous variables were evaluated using the Mann-Whitney test and were expressed as median ± interquartile range (IQR). P < 0.05 was considered statistically significant. All statistical analyses were presented using MedCalc for Windows, version 7.4.1.0 (MedCal Software, Mariakerke, Belgium).

## Results

The demographic, biochemical, and immunological characteristics of patients with PBC, other autoimmune liver diseases, ALC, and healthy controls are presented in **Table 2**.

**Table 2:** The demographic, biochemical, immunological and histological features of PBC patients and control groups.

	PBC (n=92)	Other autoimmune liver diseses (n=47)	ALC (n=15)	Healthy adult blood donors (n = 30)
Age, years	50 (27-75)	49 (22 -67)	49 (24-69)	33 (19 – 53)
Females/males	87/5	25/22	20/20	22/8
Bilirubin (Total), mg/dL	1.9 (1.9)	1.5 (2.4)	2.9 (4.5)	0.7 (0.5)
AST, U/L	88.6 (54.4)	70.8 (71.0)	83.7 (70.1)	22.5 (21.6)
ALT, U/L	92.5 (72.0)	73.3 (59.4)	57.6 (128.3)	15.1 (26.2)
AP, U/L	403.3 (377.6)	269.2 (175.4)	308.3 (196.5)	38.7 (16.8)
γ-GT, U/L	262.3 (226.2)	291.9 (204.0)	132.7 (58.7)	18.6 (4.8)
Albumin (g/dl)	3.6 (0.9)	3.1 (1.8)	2.9 (0.9)	4.5 (2.3)
γ-globulin (g/dl)	1.7 (1.1)	1.6 (1.7)	1.5 (1.8)	1.1 (0.2)
AMA M2	78 (85%)	0 (0%)	0 (0%)	0 (0%)
Anti-gp210 antibodies	30 (33%)	0 (0%)	0 (0%)	0 (0%)
Anti- KLHL12 antibodies	31 (34%)	0 (0%)	0 (0%)	0 (0%)
Anti- HK1 antibodies	27 (29%)	0 (0%)	0 (0%)	0 (0%)
Early histological stage (I/II)	60 (65%)	21 (45%)	8 (53%)	0
Advanced histological stage (III/IV)	26 (28%)	4 (21%)	4 (27%)	0
Ambiguous histological stage	6 (7%)	0	0	0

Data are presented as mean ± SD. γ-GT, γ-glutamyl transpeptidase; ALT, alanine aminotransferase; AP, alkaline phosphatase; AST, aspartate aminotransferase. Normal value: bilirubin < 1.2 mg/dL; AST < 40 U/L; ALT < 40 U/L; AP < 115U/mg/dL; γ-GT < 50 U/L; albumin 3.5-5,5 g/dL, γ-globulin.

In the studied PBC group, 87 out of 92 patients were females. The mean age at PBC diagnosis was 50 years. The total bilirubin concentration was higher in over 50% of the tested patients. We found increased activity of AP and γ-GT in over 75% of the verified samples, and the activity of AST and ALT was also higher in over 55% of them. AMA M2, specific autoantibodies for PBC, was found in 85% of patients’ sera, and specific anti-nuclear antibodies—anti-gp210 autoantibodies were noticed in 33% of patient’s sera.

### Detection of anti-bacterial antibodies

Seroprevalence of anti-*Cpn*, anti-*Y. enterolitica*, anti-*Hp*, anti-*M. pneumoniae* and anti-*E.coli* antibodies in the PBC group were significantly higher than those in pathological and healthy control and were: 74%, 40%, 84%, 39% and 69% respectively. The odds ratios (ORs) of the presence of anti-bacterial antibodies for the PBC patients versus the healthy control and *p-value* are shown in **Table 3**.

**Table 3:** Percentage of positive sera samples of patients with PBC against various infections agents.

	PBC patients (n = 92) %	Controls (n = 92) %	Odds ratio (CI ± 95%)	*p* value
Anti*-Chlamydia pneumoniae*	74	25	8.5 (4.4-16.5)	< 0.0001
Anti*-Helicobacter pylori.*	84	47	5.8 (2.9-11.6)	< 0.0001
Anti*-Mycoplasma pneumoniae*	39	18	2.8 (1.4-5.6)	0.0024
Anti*-Yersinia enterolitica.*	40	27	1.9 (1.0-3.6)	0.0430
Anti*-Chlamydia trachomatis* Anti*-E.coli*	16 69	8 18	2.4 (0.9-6.1) 6.3 (3.2-12.3)	0.0753 < 0.0001

**Figure 1:**
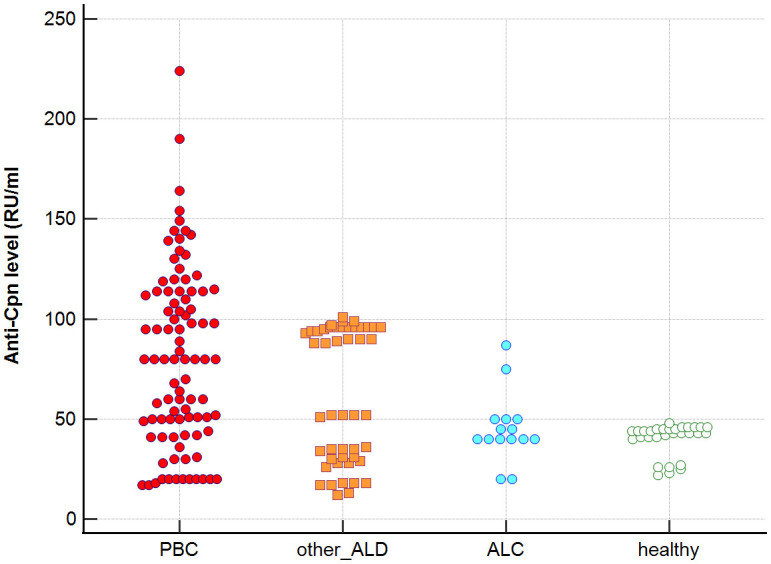
Anti-*Cpn* antibodies level in studied groups.

**Figure 2:**
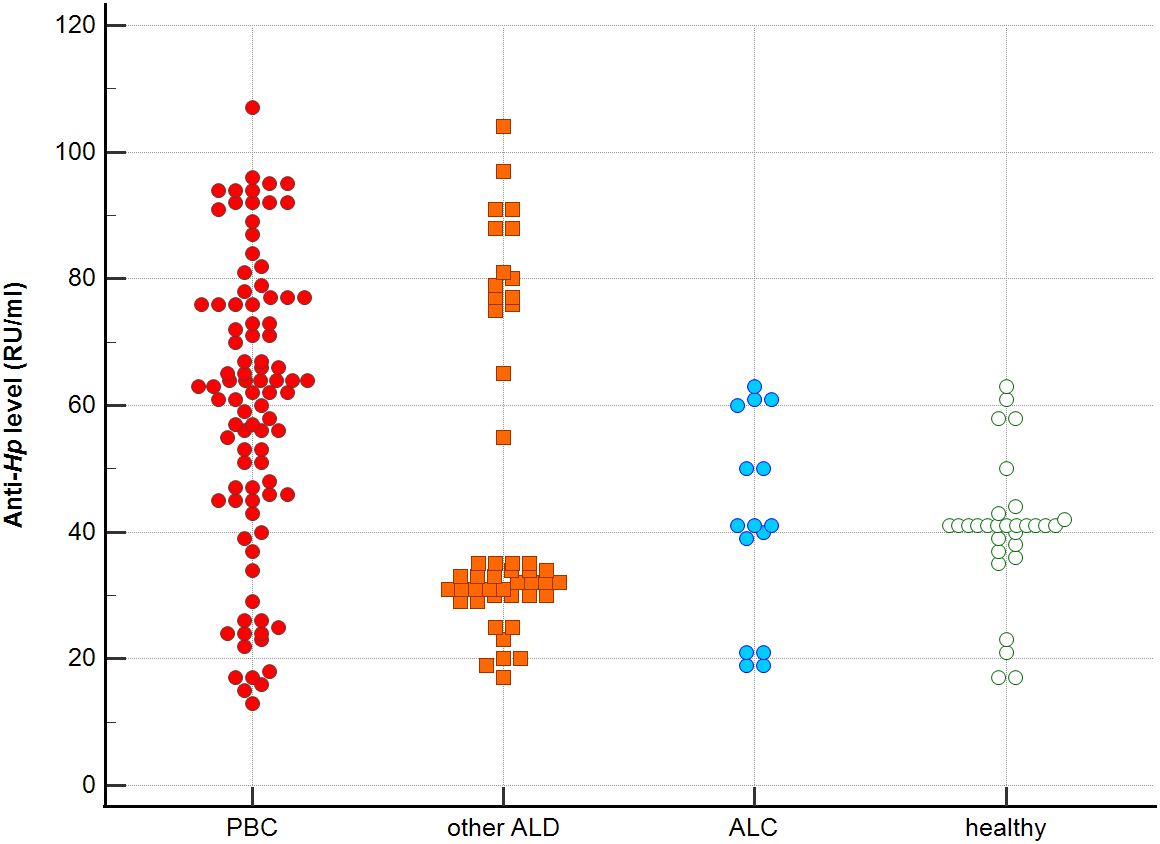
Anti-*Hp* antibodies level in studied groups.

**Figure 3:**
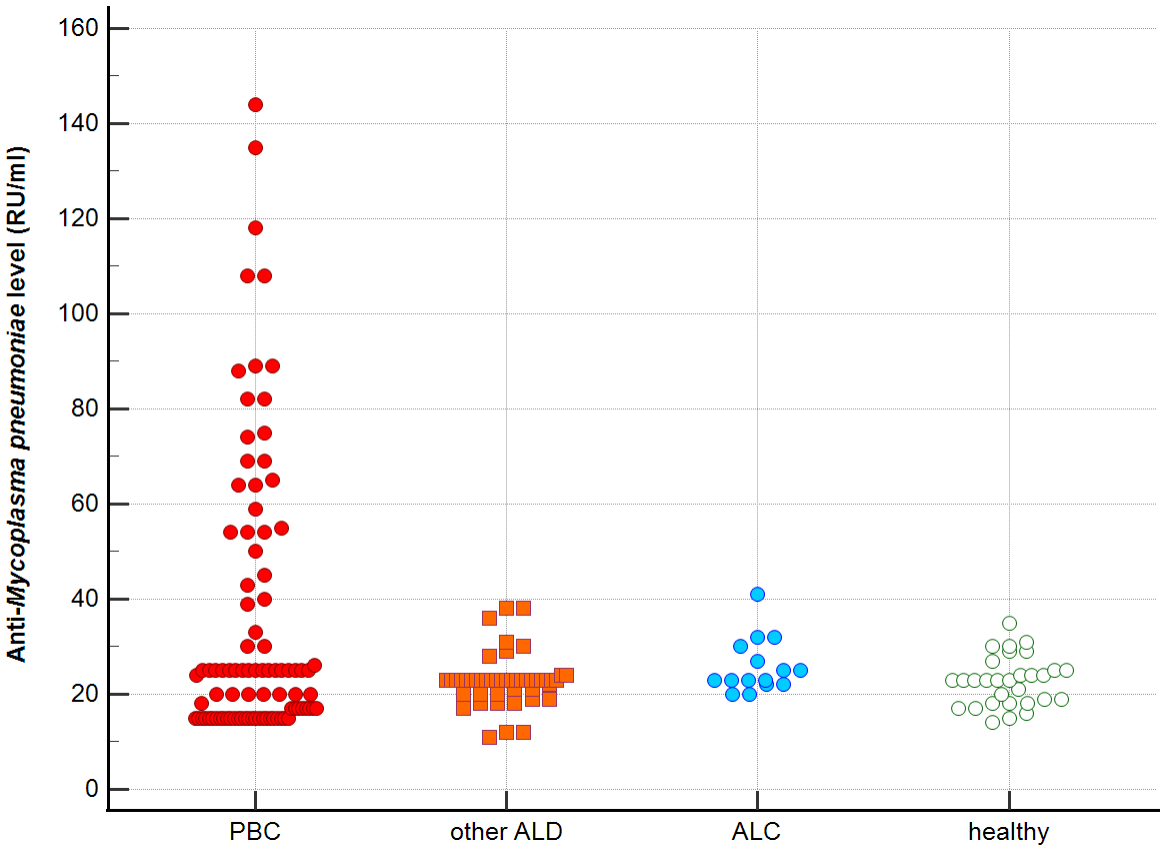
Anti-*M. pneumonia* antibodies level in studied groups.

**Figure 4:**
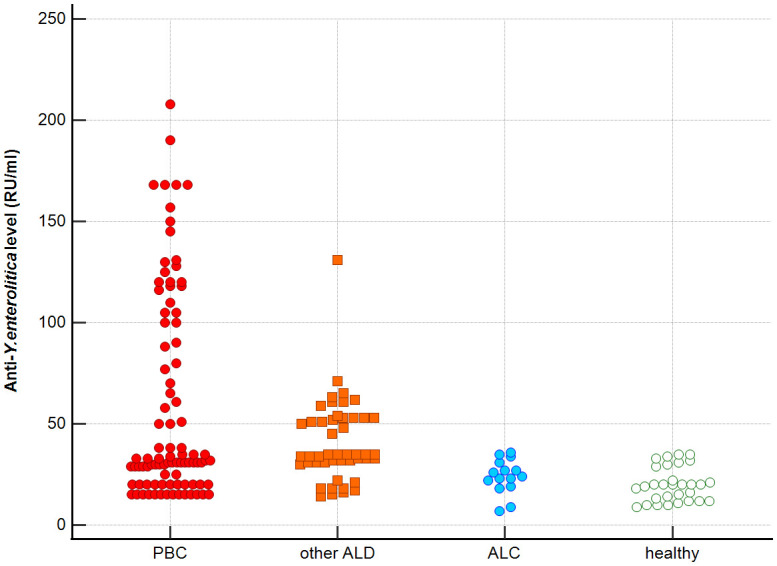
Anti-*Y. enterolitica.* antibodies level in studied groups.

**Figure 5:**
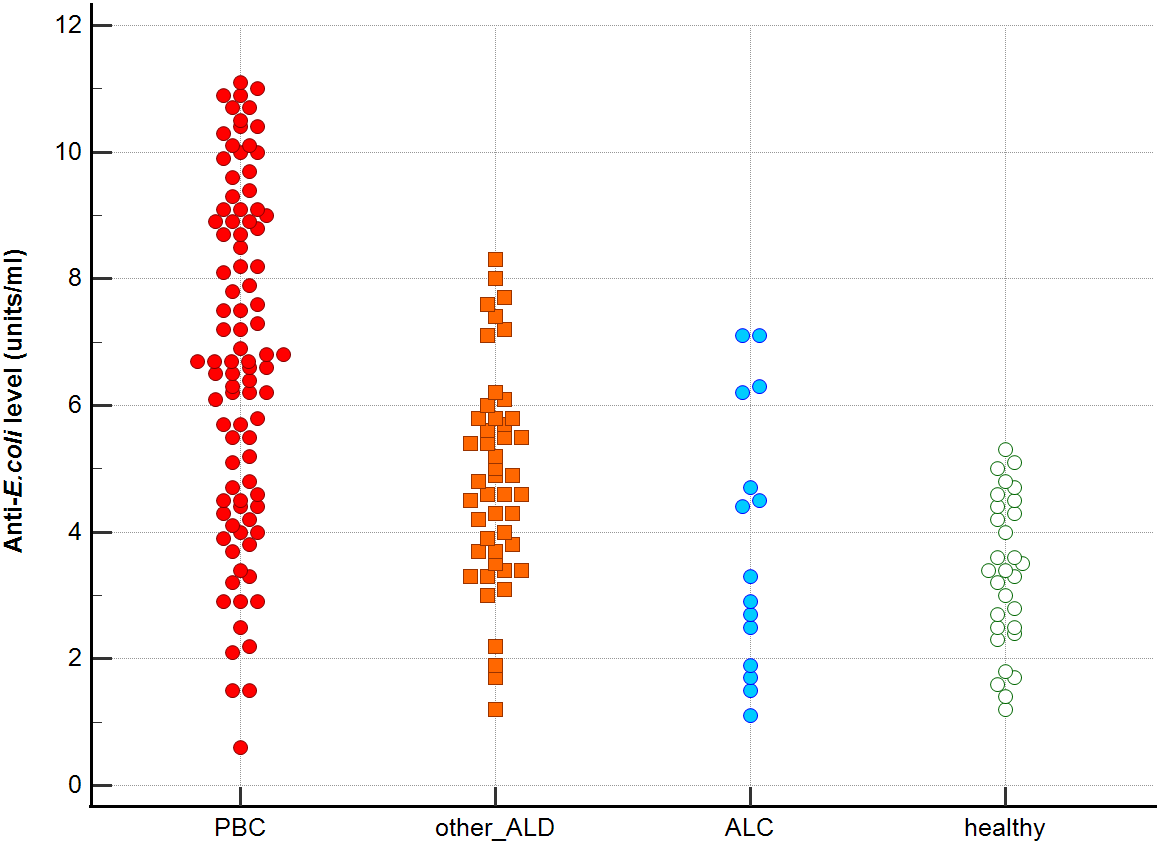
Anti-*E.coli* antibodies level in studied groups.

Distribution the antibodies to these bacterial antigens in PBC patients and the control groups is presented in **Figures 1**–**5**. The mean level of anti-*Cpn*, anti-*Hp*, anti- *M. pneumoniae*, anti-*Y. enterolitica* and anti-*E.coli* antibodies in the PBC group was significantly higher than those in the healthy group 80 ± 44 *vs*. 43 ± 10 RU/ml, p < 0.0001; 60 ± 22 *vs.* 41 ± 10 RU/ml, p < 0.0001; 36 ± 30 *vs.* 23 ± 5 RU/ml; *p =* 0.020 and 59 ± 50 *vs.* 20 ± 8 RU/ml, p < 0.001, 6.8 ± 2.6 *vs.* 3.3 ± 1.1 units/ml, p < 0.0001, respectively (**Supplementary Figures 1-5**).

The mean level of antibodies directed against bacterial antigens in sera of patients with PBC was also higher than in alcoholic liver cirrhosis: 80 ± 44 vs. 45 ± 17 RU/ml, p < 0.0001 for anti-*Cpn;* 60 ± 33 *vs.* 41 ± 16 RU/ml*, p* = 0.003 for anti-*Hp*; 36 ± 30 vs. 23 ± 5 RU/ml; p = 0.059 for anti- *M. pneumoniae*, 59 ± 50 vs. 20 ± 8 RU/ml, p < 0.0001 for anti- *Y. enterolitica*, and 6.8 ± 2.6 *vs.* 4.0 ± 2.1 units/ml, p = 0.0001for anti-*E.coli* (**Supplementary Figures 1-5**).

The mean level of antibodies directed against bacterial antigens in sera of patients with PBC was also elevated than in other liver diseases with a significant difference: 80 ± 44 vs. 62 ± 15 RU/ml, p < 0.0001 for anti-Cpn; 60 ± 22 vs. 45 ± 26 RU/ml, p < 0.0001for anti-Hp; 36 ± 30 vs. 26 ± 7 RU/ml; p = 0.059 for anti- *M. pneumoniae*, 59 ± 50 vs. 41 ± 20 RU/ml, p < 0.0001 for anti- *Y. enterolitica* and 6.8 ± 2.6 *vs.* 4.9 ± 1.7 units/ml, p < 0.0001 for anti-*E.coli* (**Supplementary Figures 1-5**).

Significantly lower concentrations ​​of antibodies against bacterial antigens were observed in the sera of patients with other autoimmune diseases, in all cases except anti-*M. pneumoniae* antibodies, the differences compared to the healthy group were also statistically significant: p < 0.0030 for anti-*Cpn* antibodies, p < 0.001 for anti- Y. *enterolitica* antibodies, p =0.018 for anti*-Hp*, p = 0.499 for anti-*M. pneumoniae* and *p* < 0.0001 for anti-*E.coli* antibodies (**Supplementary Figures 1-5**).

The concentration of antibacterial antibodies in the sera of patients with ALC was at the level of the healthy group, and no significant statistical difference was found: p = 0.584 for anti-*Cpn* antibodies, p = 0.099 for anti- *Y. enterolitica* antibodies, p =0.694 for anti-*Hp*, p = 0.276 for anti- *M. pneumoniae* and *p* = 0.148 for anti- *E.coli* antibodies (**Supplementary Figures 1-5**).

**Figure 6:**
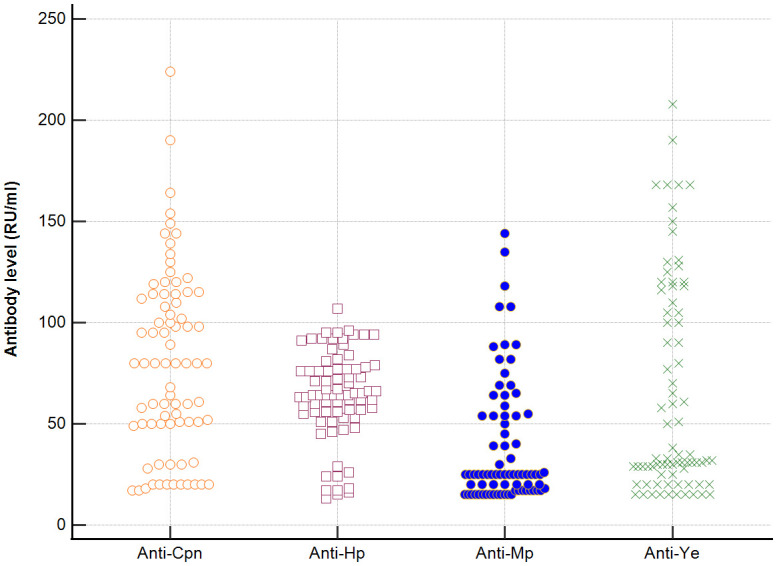
Distribution of the antibodies to the bacterial antigens in PBC AMA M2 positive patients.

**Figure 7:**
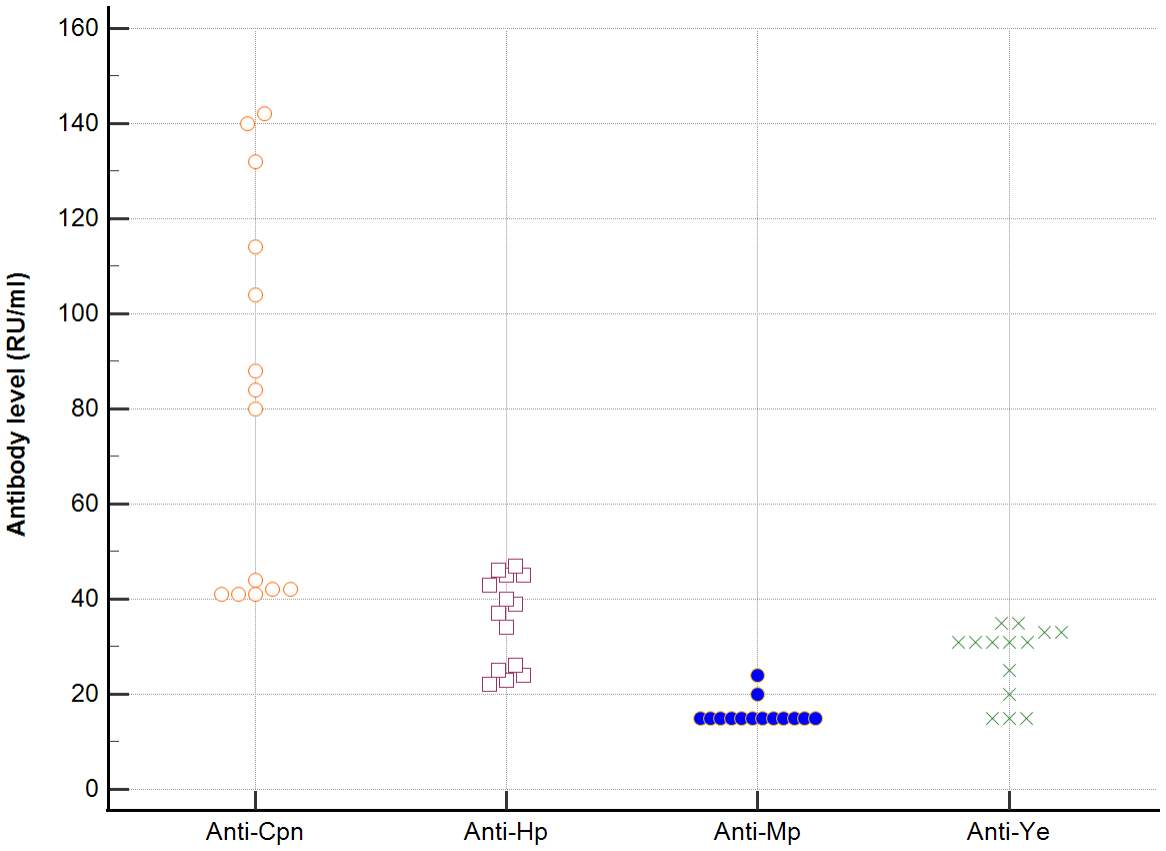
Distribution of the antibodies to the bacterial antigens in PBC AMA M2 negative patients.

**Figure 8:**
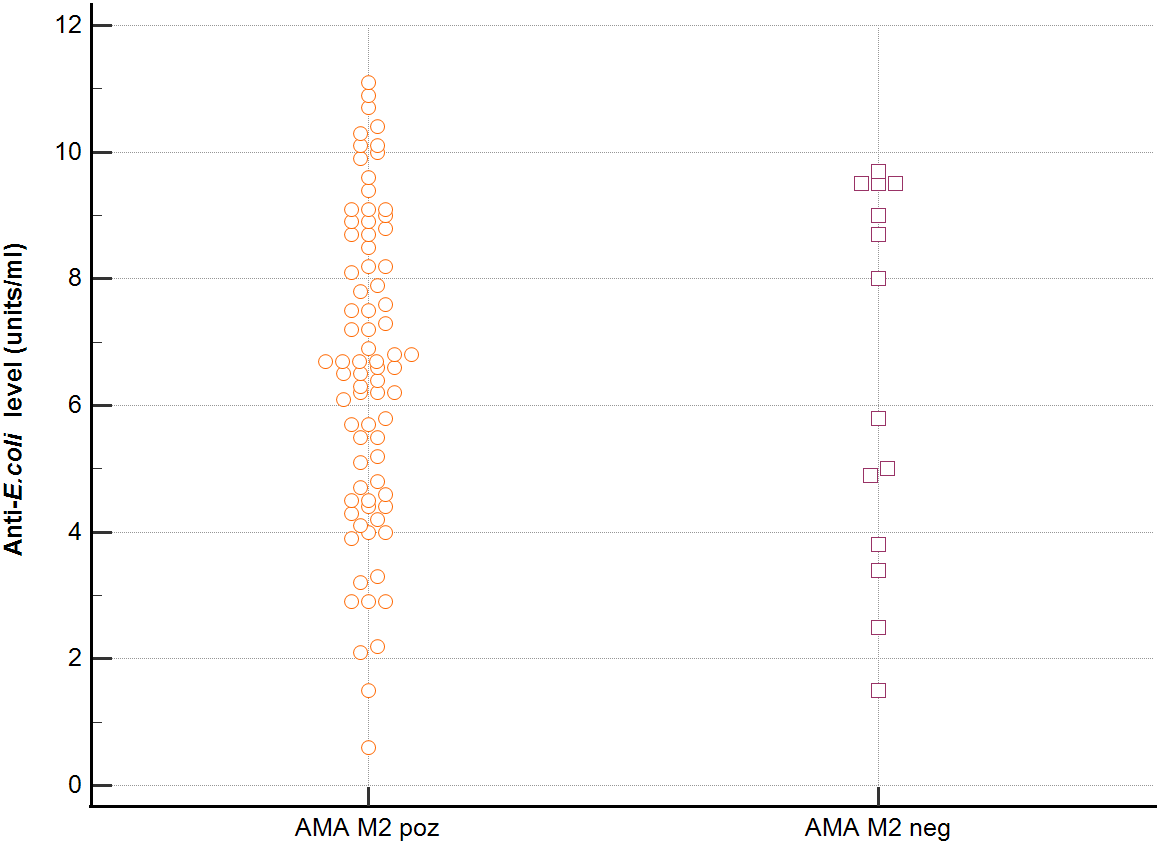
Anti-*E.coli* antibodies level in the PBC AMA M2 positive and AMA M2 negative patients.

In sera of patients with positive AMA, specific for PBC, antibodies directed against bacterial antigens have been found in 80% vs. 50% in sera with AMA negative. Distribution of the antibodies to these bacterial antigens in PBC AMA M2 positive and AMA M2 negative patients has been shown in **Figures 6**–**8**. The mean level of, anti-*Hp, M. pneumoniae and* anti- *Y. enterolitica* antibodies in the PBC AMA M2 positive group was significantly higher than those in the AMA M2 negative group 64 ± 20 *vs*. 35 ± 10 RU/ml, p < 0.0001; 40 ± 31 *vs.* 16 ± 3 RU/ml, p = 0.005 and 65 ± 53 *vs.* 27 ± 8 RU/ml; *p =* 0.009, respectively. However, the level of antibodies against *Cpn* and *E.coli* in both groups was not statistically different.

Seroprevalence of anti-*Ch. pneumoniae* antibodies and anti-*Hp* antibodies were definitely higher in PBC patients with AMA positive than in PBC patients without AMA, p=0.047 and p=0.038, respectively. Among patients with AMA-negative PBC, the presence of anti-*Ch. pneumoniae* antibodies were found in 50% and anti-*Hp* antibodies were found in 63% of patients (**Supplementary Figures 6, 7**). Anti-*M. pneumoniae* antibodies and anti-*Y. enterolitica.* antibodies were positive only in PBC patients with AMA positive, p=0.022 (**Supplementary Figures 8, 9**) In PBC sera of patients with positive AMA, antibodies directed against *E.coli* antigen have been found in 74% vs. 50% in sera with AMA negative.

We evaluated the correlation of individual patient values against each other shown them in **Table 4**. The levels of AMA M2 antibodies versus the levels of anti-*Cpn*, AMA M2 antibodies versus the levels of anti-*Y. enterolitica*, AMA M2 antibodies versus *anti-Hp*, AMA M2 antibodies versus anti-*M. pneumoniae* and AMA M2 versus anti-*E.coli*. Also mutual relationship between these measured antibacterial antibodies has been presented (**Table 4**).

**Table 4:** Relationship between measured antibacterial antibodies and AMA M2 antibodies.

	Anti-*Ch. pneumoniae*	Anti-*H. pylori*	Anti-*M. neumoniae*	Anti- *Y. enterolitica*	Anti- *E. coli*
**AMA M2**	r = 0.28 *p* = 0.017	r = 0.51 *p* < 0.001	r = 0.69 *p* < 0.001	r = 0.61 *p* < 0.001	r = 0.45 *p* < 0.001
**Anti- *Ch. pneumoniae***		r = 0.40 p < 0.001	r = 0.23 *p* < 0.003	r = 0.20 *p* < 0.061	r = 0.58 *p* < 0.001
**Anti-*H. pylori***			r = 0.52 *p* < 0.001	r = 0.39 *p* < 0.001	r = 0.33 *p* < 0.001
**Anti- *M. pneumoniae***				r = 0.65 *p* < 0.001	r = 0.28 *p* < 0.008
**Anti- *Y. enterolitica***					r = 0.18 *p* < 0.080

### Detection of inhibition of antibody-antigen binding

**Figure 9:**
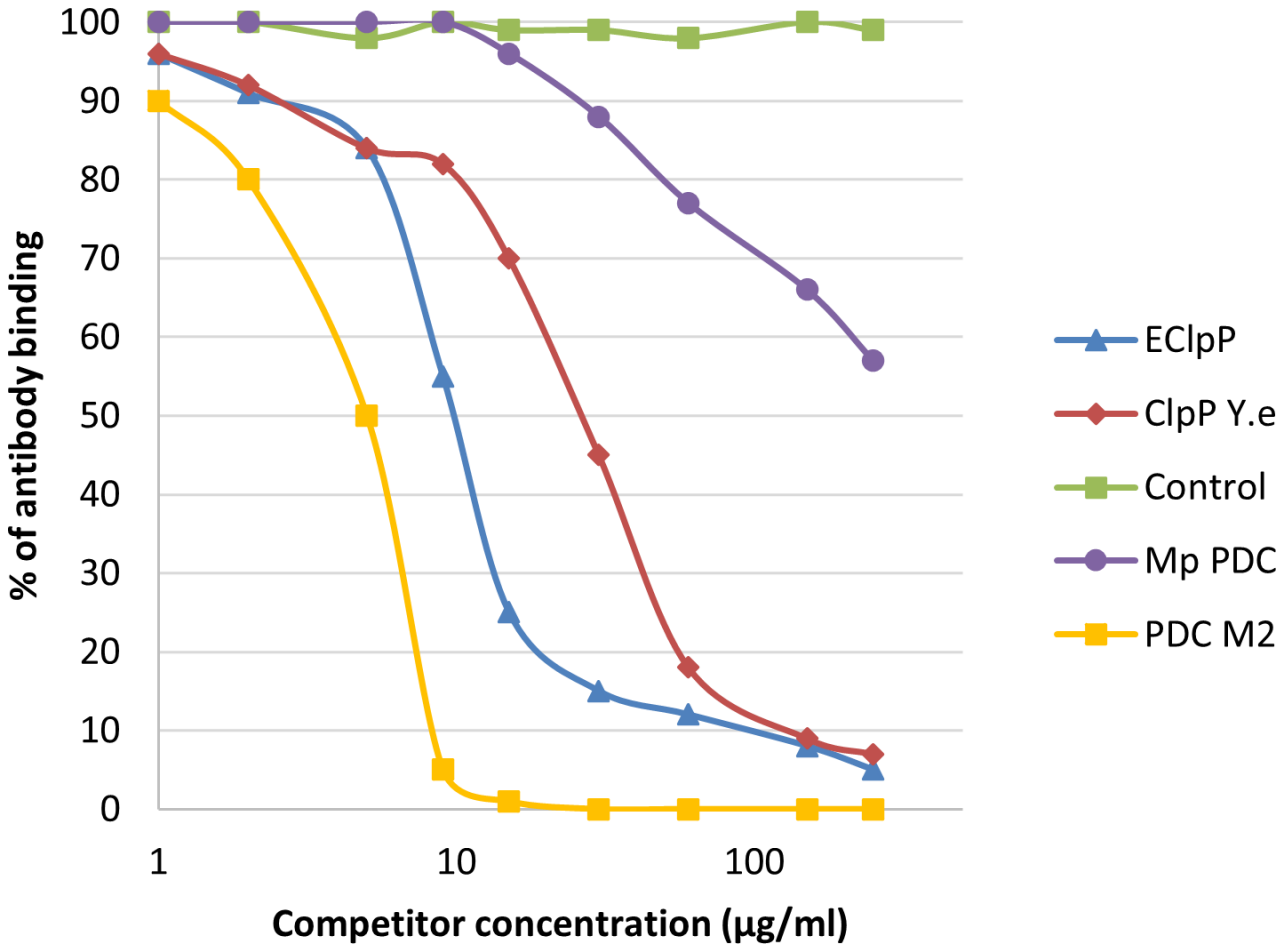
Inhibition of antibody binding to human PDC-E2 by bacterial peptides; *ECLpP, E. coli caseinolytic protease P; ClpPYe*, peptide of *Yersinia enterolitica; Mp* PDC – *Mycoplasma pneumoniae* peptide.

**Figure 10:**
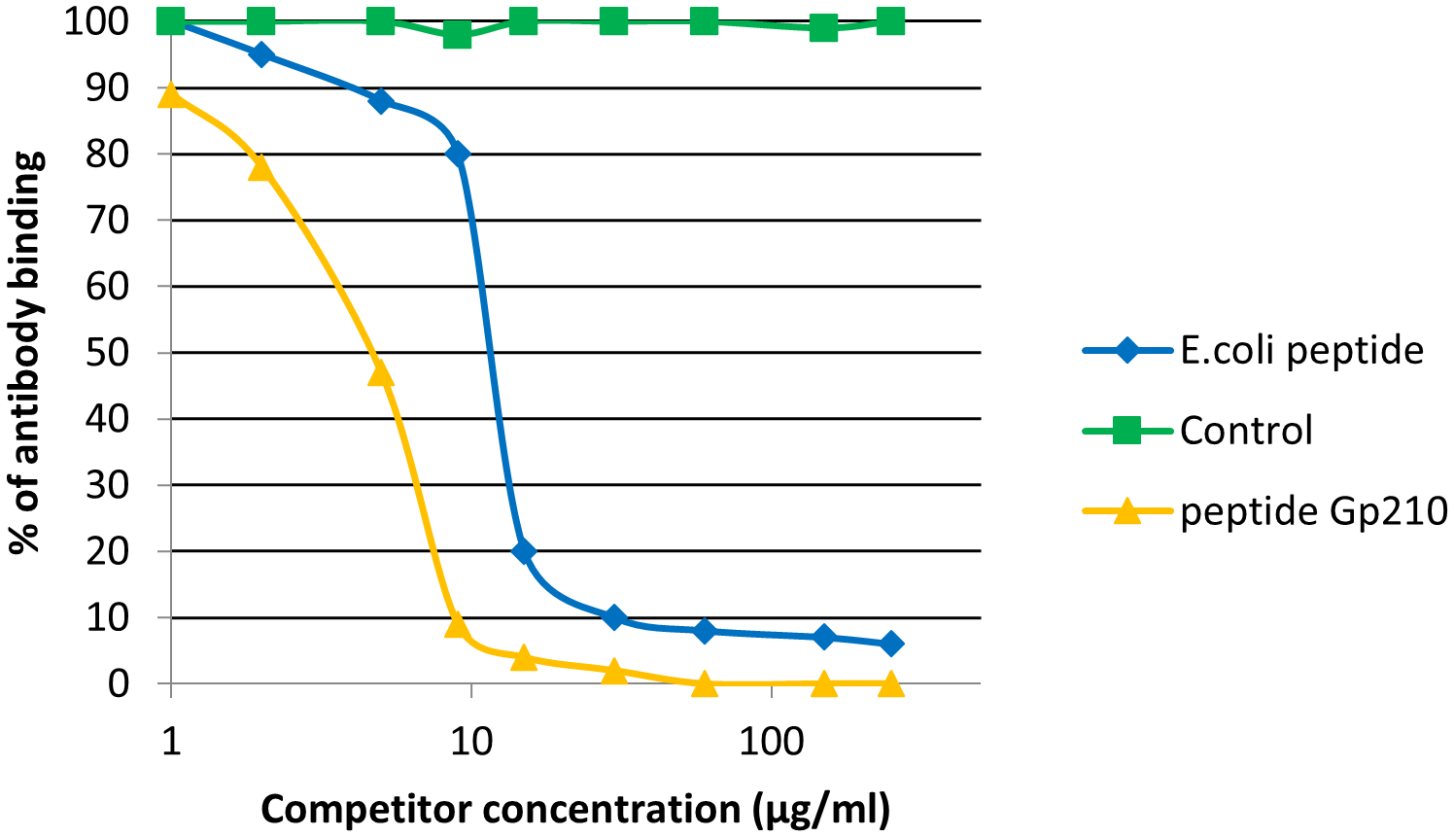
Inhibition of antibody binding to human gp210 antigen by bacterial peptides *E. coli* peptide - Adenine glycosylase of *E. coli* - QRPPSGLWGGLY, Gp210 antigen – DRKASPPSGLWSPAYASH.

We noticed inhibition of specific antigen-antibody binding to the human PDC M2 antigen by the bacterial peptides: EClpP and adenine glycosylase from *E. coli* caseinolytic protease P, ClpP *Y.e* from the peptide of *Y. enterolitica* and *Mp* PDC from *M. pneumonia* peptide (**Figure 9**). Reactivity to the EClpP peptide was detected in 39 out of the 92 sera PBC patients (42%), to the ClpP *Y.e* was determined in 28 out of 92 sera PBC patients (30%). The immunological activity of bacterial peptides expressed as the amount of protein needed to inhibit 50% of the binding of autoantibodies to the human PDC M2 antigen were 12 µg/mL for EClpP and 30 µg/mL for ClpP *Y.e*. The inhibition of autoantibody-antigen binding by *Mp*PDC was much weaker. For the bacterial peptides *E.coli* peptide - adenine glycosylase of *E.coli* amount of protein necessary to obtain 50% inhibition of autoantibody binding to the gp210 antigen was 14 µg/mL (**Figure 10**).

**Figure 11:**
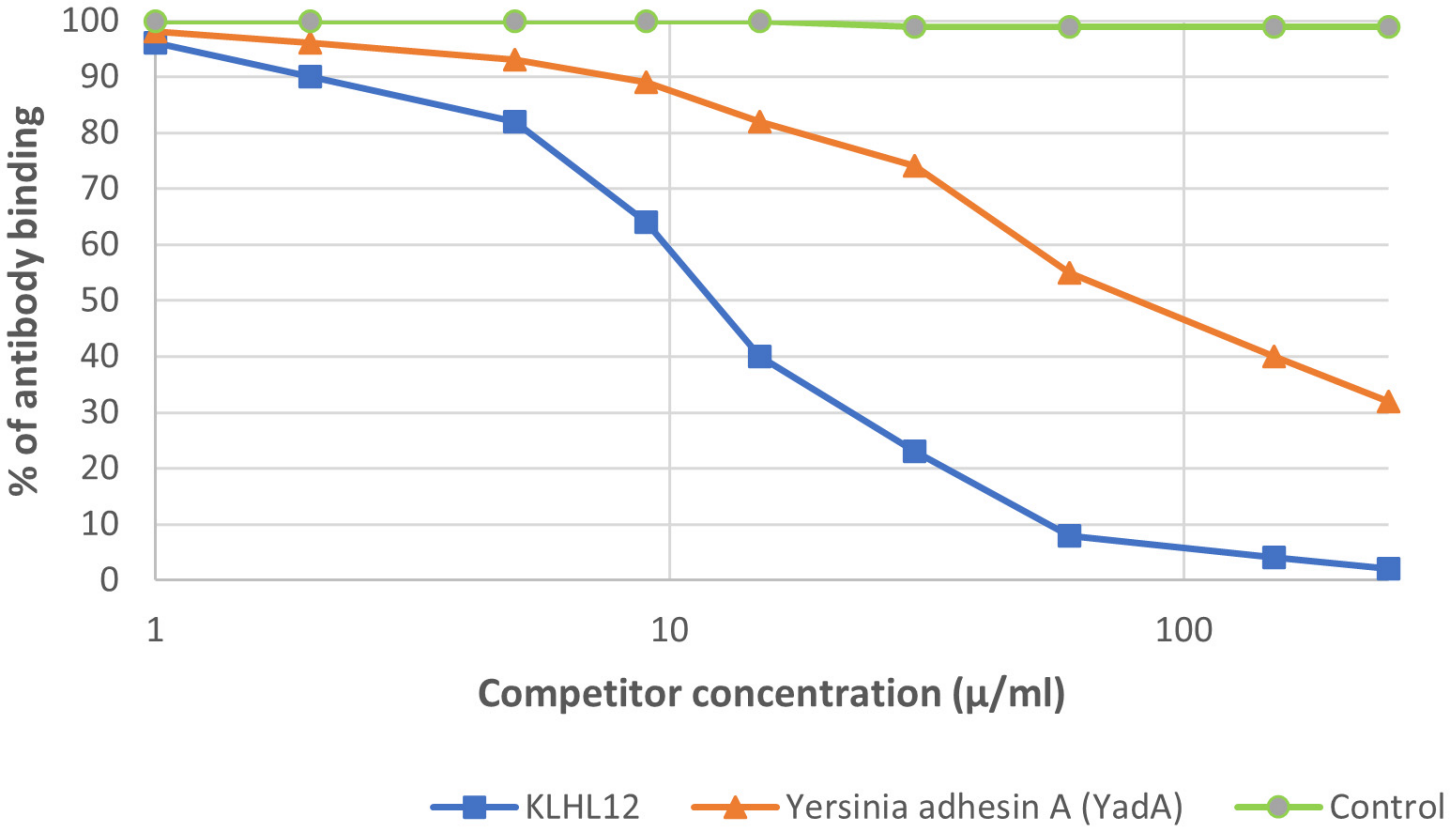
Inhibition of antibody binding to human KLHL12 protein by bacterial peptide of Yersinia enterolitica - Yersinia adhesin A (YadA).

We observed inhibition of specific antigen-antibody binding to the human KLHL12 protein by the bacterial peptide: adhesin A (*Y*adA) from *Yersinia* (**Figure 11**). Reactivity to the *Y*adA peptide was detected in 27 out of the 92 sera PBC patients (29%). The immunological activity of bacterial peptides expressed as the amount of protein needed to inhibit 50% of the binding of autoantibodies to the human KLHL12 antigen was 70 µg/mL.

We studied also, the inhibition of antibody binding to human HK-1 protein by bacterial peptide – caseinolytic protease subunit X of *borrelia Burgdorferi* and we observed the immunological activity of this bacterial peptides – the inhibition 50% of the binding of autoantibodies to the human HK1 antigen was 150 µg/mL.

## Discussion

Environmental factors‐mediated activation of autoimmune response and subsequent autoimmunity disease, including PBC could be caused through molecular mimicry, bystander activation, or both (Principi et al., 2017; Tanaka et al., 2019). The phenomenon of molecular mimicry includes in its structure the similarity of the antigenic determinants of the causative agent (microbial, xenobiotic) to the epitopes of some host proteins, which initiates an immunological cross-reaction. There is an association between PBC and exposure to specific infections (Münz et al., 2009). It is supposed that the development of AMA in PBC may be the effect of contact with infectious agents. Kikuchi et al. incubated peripheral blood mononuclear cells of PBC patients with short non-protein CpG-DNA sequences (unmethylated cytosine-phosphate-guanine - CpG motifs), commonly present in the cells of various bacteria. An increase in the number of activated B lymphocytes was found in PBC patients (Kikuchi et al., 2005).

We tried to determine the association between tested infectious agents and AMA status, in the light of the mechanism of cross-reactivity of microbial and human PDC-E2. We studied whether the presence of anti-infectious agents - Abs found in high frequency in Polish PBC patients (and their co-occurrences) correlates with the presence of anti-mitochondrial antibodies. The prevalence of anti-infectious antibodies was significantly elevated among our PBC patients when compared with controls. We observed also high levels of anti-*Ch. pneumoniae*, anti-*Y. enterocolitica*, anti-*H. pylori*, anti- *M. pneumoniae* and *E.coli* antibodies in sera of patients with PBC. But reactivity was also detected in the sera of AMA-negative PBC patients, while control sera did not react against bacterial proteins. Anti-*M. pneumoniae* antibodies and anti-*Y. enterolitica*. antibodies were positive only in PBC patients with AMA positive, but anti-*Ch. pneumoniae* antibodies and anti-*H. pylori* antibodies we determined also in the sera of AMA-negative PBC patients. Tanaka et al. also made similar observations (Tanaka et al., 2019). Jun Zhang et al. found high levels of autoantigen-specific peripheral plasma blasts, suggesting activation of memory B cells (Zhang et al., 2014).

*Cpn* plays a role in autoimmune diseases such as multiple sclerosis or primary sclerosing cholangitis (Layh-Schmitt et al., 2000; Ponsioen et al., 2002). A high *Cpn* IgG level indicates a past infection. The study of Lamacchia et al. describes a potential role of past *chlamydial* infection in the development of RA (Rheumatoid arthritis) (Lamacchia et al., 2023). In our study, about 70% of PBC patients have anti-*Cpn* antibodies. Their occurrence and mean levels were higher than in healthy and pathological controls. This is why it could be concluded that *Cpn* infection could be a potential cause of PBC. Hai-Ying Liu et al. also reported that up to 68.3% PBC patients were infected with *Cpn* (Liu et al., 2005). The first study showing that *Ch. pneumoniae* antigens were noticed more frequently in the livers of PBC patients compared to control groups was performed by Abdulkarim et al. (Abdulkarim et al., 2004).

We have shown that there may be a relationship between PBC and a form of chronic *Y. enterocolitica*. Anti-*Y. enterocolitica* antibodies were positive only in PBC patients with AMA positive. We noticed inhibition of specific antigen-autoantibody binding by the bacterial ClpP from *Y. enterolitica*. Research on the influence of *Y. enterolitica* on the development of PBC has already been undertaken by Roesler et al. (Roesler et al., 2003). The authors did not demonstrate that this bacterial factor triggers PBC. They attempted to identify the β-subunit of bacterial RNA-Polymerase—a non-species-specific bacterial protein as a target of antibodies in PBC and rather suggested that antibodies to the β-subunit of bacterial RNA polymerase belong to the pool of natural antibodies, the level of which can be elevated in autoimmune liver diseases. A little earlier the PBC-specificity of anti-microbial ClpP reactivity was confirmed by Bogdanos et al (Bogdanos et al., 2002). Fang et al. discussed the recent data on how *Y. enterocolitica* may link to the pathogenesis of Crohn’s disease (Fang et al., 2023).

Positive anti-*M. pneumoniae* antibodies we found also only in PBC patients with AMA positive. The mean level of antibodies directed against these bacterial antigens in sera of patients with PBC was also higher than in other studied groups with a significant difference. *Mycoplasma* antigens as a probable trigger for the induction of antimitochondrial antibodies in PBC was also found by Berg et al. (Berg et al., 2009). They concluded that patients with PBC demonstrate a higher occurrence of *Mp* PDC-E2-related antibodies and, molecular mimicry between surface molecules of *mycoplasma* and epitopes of the autoantigen can play a main role in the etiopathology of PBC. In turn, Iwasa et al. studied autoimmune neurological diseases and found that the difference of anti-acetylcholine receptor antibody titer in myasthenia gravis is connected to incidence of *M. pneumoniae* and influenza virus (Iwasa et al., 2018). The level of antibodies against *Y. enterolitica* and *M. pneumoniae* correlated best with the level of AMA M2 antibodies in the sera of our patients.

The work of Abenavoli et al. suggests a pathogenetic role of increased intestinal permeability in the course of *H. pylori* infection and the role of infection in the development of PBC (Abenavoli et al., 2010). Perhaps *H. pylori* penetrates the bile ducts, causing some predisposed patients to develop an autoimmune disease. Wang et al. provide the latest review of current support or opposition for *H. pylori* as a factor in autoimmune diseases, including autoimmune liver diseases (Wang et al., 2022). The mitochondrial autoepitopic area of pyruvate dehydrogenase complex E2 (PDC-E2) has been similar to urease β of *H. pylori*, which suggests that *H. pylori* infection can be linked to the prevalence of PBC. Waluga et al. reached similar conclusions (Waluga et al., 2015). The presence of *Hp* DNA in liver tissue, and antibodies against the microorganism in the bile and serum of PBC patients were also discovered by Bogdanos et al (Bogdanos and Vergani, 2009). There are also more studies on the intestinal microbial flora, containing more genes than the human genome, which may be an important factor inducing diseases on the intestinal-liver axis, also in the case of *Hp* (Kummen et al., 2017; Adolph et al., 2018; Tang et al., 2018; Wei et al., 2020). Only a few studies have focused on AIH. *H. pylori* DNA was determined in the liver tissues of some patients with AIH, but no significant differences were noticed in the comparison between these patients and controls (Smyk et al., 2014). It is known that *H. pylori* can affect the intestinal microflora and can also cause the translocation of pathogens, which in turn may promote the development of PBC. Kitahata et al. discovered dysbiosis in the mucosa-associated microbiota profile of the small intestine in patients with PBC and typical bacterial overgrowth that has been reported to be associated with PBC (Kitahata et al., 2021). Work by Eamonn and Quigley presents epidemiological, clinical and some experimental evidence for the role of bacteria in the pathogenesis of PBC (Eamonn and Quigley, 2016). Currently, there is little literature on the impact of gut microbiota on PBC and further research is needed. Works continuing this topic could seem very interesting. In our serological diagnosis, seroprevalence of anti-*Hp* antibodies in the PBC group were significantly higher than those in healthy control, lower concentrations of antibodies against *Hp* antigen were observed in the sera of patients with other autoimmune diseases, also with statistically significant difference. AMA positive and AMA negative PBC patients’ sera have been tested for antibodies against multiple infectious agents, including *h. pylori* by Shapira et al. (Shapira et al., 2012). They also observed, that the prevalence of anti-*H. pylori* antibodies was significantly elevated among PBC patients when compared with controls. Finally, no differences they observed between AMA negative sera and their AMA positive counterparts with regard to seroprevalence of the investigated infectious agent, but they suggested that exposure to infectious agents may contribute to PBC risk.

The relationship between among others infection, like vaginal infection and PBC was studied by Gershwin et al (Gershwin et al., 2005). They showed a higher incidence of PBC associated with a history of vaginal infection (*P* value = 0.0018). However, only UTI, no vaginal infection has been presented as a possible risk factor at PBC. Additionally, we also checked the presence of antibodies against *Chlamydia trachomatis*. We did not find any significant statistical differences, so we did not take this infectious agent into explanation in the next research. The pathogen that is detected in most UTI patients is *E. coli.* Tanaka et al. and Wang et al. discovered that *E. coli* infection may lead to the recognition of self-antigens by autoreactive T cells and B cells (Wang et al., 2014; Tanaka et al., 2019). An *E. coli* infection, may be associated with PBC through molecular mimicry between human and bacterial PDC-E2. Floreani et al. observed that *E. coli* infection may be a factor that breaks the immunological tolerance to the mitochondrial autoantigen, resulting in the production of AMA (Floreani et al., 2016). PDC-E2 is highly conserved in both prokaryotes and higher organisms. *E. coli* PDC-E2 titers in the serum of some patients were lower, and in others, they appeared later in the disease than human PDC-E2. Therefore, it seems that other bacteria may also be involved in the etiopathogenesis of PBC, also through molecular mimicry (Tanaka et al., 2019). The cross-reaction of antibodies against the human mitochondrial pyruvate dehydrogenase complex with the *E. coli* pyruvate dehydrogenase complex in PBC has already been reported by Bogdanos et al. (Bogdanos et al., 2004). Hou et al. presented the TCRβ repertoire of memory T cells and suggested a potential role of Escherichia coli in the pathogenesis of primary biliary cholangitis (Hou et al., 2019). The bacterial peptides of *E.coli*, *Yersinia enterolitica* and *M. pneumoniae* with a similar structure to human PDC-E2 react with sera from patients with PBC. We have demonstrated that PDC-E2 cross-reacts with homologous sequence in *E. coli* PDC-E2. Recent studies demonstrate that contact with *E. coli* induces specific antibodies against *e*PDC-E2, resulting in determinant spreading and the development of classic autoantibodies, leading to the development of PBC (Yang et al., 2022). We also observed the influence of antibody binding to PDC-E2 and gp210 antigens by bacterial agents. The level of anti-E.coli antibodies in patients with AMA M2 positive and AMA M2 negative PBC was not characterized by a significant statistical difference, therefore it seems that molecular mimicry between the bacterial antigen and the specific gp210 antigen may play an additional role.

We have included to the manuscript our pilot studies on possible molecular mimicry between the sequence of PBC-specific antigens discovered a few years ago (KLHL12 and HK1) and bacterial pathogens. We explored novel protein candidates in *Yersinia enterolitica* - adhesin A, based on probability of molecular mimicry between *Y*adA (NSVAIGXXS) and KLHL12 (SVAPMNURRG) and caseinolytic protease subunit X of *borrelia Burgdorferi* based on probability of molecular mimicry between this bacterial pathogen and HK1. Future studies are needed to clarify this link, because no research has yet been conducted on the relationship between these PBC-specific antigens and infectious agents.

We also read interesting articles discussing autoimmune diseases following viral infections. Among other things, there have been works on autoimmune hepatitis after infection with the SARS-CoV-2 virus (Kabaçam et al., 2021; Marabotto et al., 2021). The authors suggest that an increase in the level of inflammatory cytokines, including INF-α (tumor necrosis factor-α), INF- γ (interferon-γ), and IL-10 (interleukin-10), after SARS-CoV-2 infection initiates autoimmune disease (Rodríguez et al., 2020). Marabotto et al. and Boettler et al.and Lee et al. showed that SARS-CoV-2-specific CD8+ T cells can cause AIH-like conditions after infection (Marabotto et al., 2021; Boettler et al., 2022; Lee et al., 2022). However, there are no data related to the development of primary biliary cholangitis after SARS-CoV-2 infection, but it seems that a similar mechanism may occur. The interaction between bacteria and host cells also modulates the immune system and results in the release of one or more cytokines. *Chlamydia pneumonia* for example might be a factor initiating the cytokine cascade in PBC. The bacterium may infect and live in number of the host cells (including circulating monocytes, tissue macrophages, or endothelial cells). If human immune system is unable to eliminate *Chlamydia pneumoniae*, the inflammation progresses to a self-supporting chronic process, in which are essentially involved macrophages infiltrating also liver tissue, increased synthesis of the pro-inflammatory cytokines. *H. pylori* having cag PAI stimulate epithelial cell lines to secrete large amounts of the pro-inflammatory cytokine IL-8. *H. pylori* infection increases the concentration of many pro-inflammatory cytokines, including IFN-γ TNF-α, IL-1β, IL-6, IL-7,IL-8,IL-12 and IL-18. The release of chemoattractants contributes to the infiltration of immune cells, especially neutrophils, which contributes to the exacerbation of inflammatory processes and cell destruction by free oxygen radicals (Moyat and Velin, 2014). In pulmonary infection, large numbers of neutrophils and lymphocytes accumulate in the alveolar fluid. CD4+ cells, B lymphocytes and plasma cells infiltrate the lungs. Immune response is a consequence of lymphocyte proliferation, Ig production, secretion of TNF-α, IFN-γ and interleukins (Mettelman et al., 2022). The release of pro-inflammatory cytokines in M. pneumoniae infections causes exacerbation of the chronic disease process in the lungs or contributes to a weakened immune response, resulting in epitope spreading to other organs. In epitope spreading, the immune response to a persisting pathogen, or direct lysis of self-tissue by the persisting pathogen, causes damage to self-tissue. Antigens released from damaged tissue are taken up by APCs, and this initiates an immune response directed towards self-antigens.

Our study also has some limitations. We attempted to link the concept of molecular mimicry in autoimmune development to *M. pneumoniae* and *Y. enterolitica*, observing positive antibody results only in AMA-positive PBC patients. Really, AMA-positive patients (85%) constitute a significant portion of PBC cases. The observed positivity rates for these antibodies (39% for *M.p*, 40% for *Y. e*) among PBC patients do not exceed half of the AMA-positive population. It would be interesting to study the presence of these antibodies in a larger population of AMA negative PBC patients, but it is very difficult to collect a large group of such patients. The scattered antibody levels anti-*Mycoplasma* pneumoniae level in studied group may suggest the need for cautious interpretation of the findings. Unfortunately, we did not have the opportunity to histologically evaluate liver biopsies from our patients for the presence of bacterial antigens. It would certainly be interesting to compare the serological results with the presence of pathogens in the biopsy sample. But on the other hand, our intention was to focus on non-invasive methods and material available after basic tests laboratory tests assessing the condition of the liver in PBC (biochemistry, autoantibodies), such as serum. We wanted to obtain as much information as possible from the patient’s serum.

In conclusion, as previously suggested, PBC is an autoimmune disease influenced by both genetic predisposing factors and environmental factors. It is very likely that these factors may not only vary from patient to patient, but may also be related to geographic location. What is new is that Polish patients were tested; previously, such tests had not been carried out in Poland. In a group of Polish patients with PBC, bacterial infection, especially *E. coli* infection, but also *Ch. pneumoniae*, *Y. enterolitica*, *H. pylori*, and *M. pneumoniae* infections may be also associated with the development of autoimmune disease through molecular mimicry between human and bacterial proteins

## Data Availability

The raw data supporting the conclusions of this article will be made available by the authors, without undue reservation.
