# Supplementary material for: Antibodies directed against bacterial antigens in sera of Polish patients with primary biliary cholangitis
Source: Front Cell Infect Microbiol. 2025 Jan 7;14:1410282. doi: 10.3389/fcimb.2024.1410282 (PMC11752878; doi:10.3389/fcimb.2024.1410282)
Supplement: Supplementary file 1 [file Table1.docx]

Supplementary Material


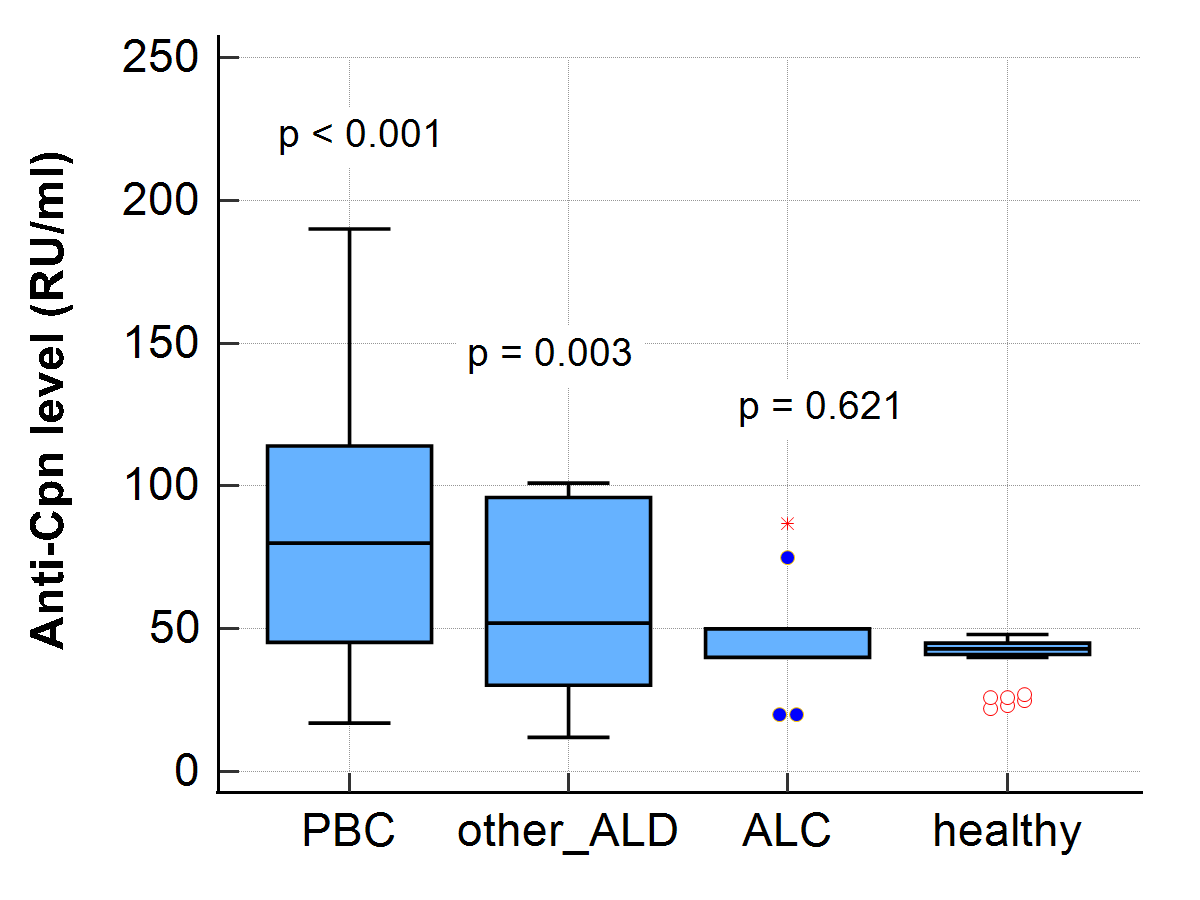


**Supplementary Figure 1.** Mean level of anti-*Cpn* antibodies in studied groups


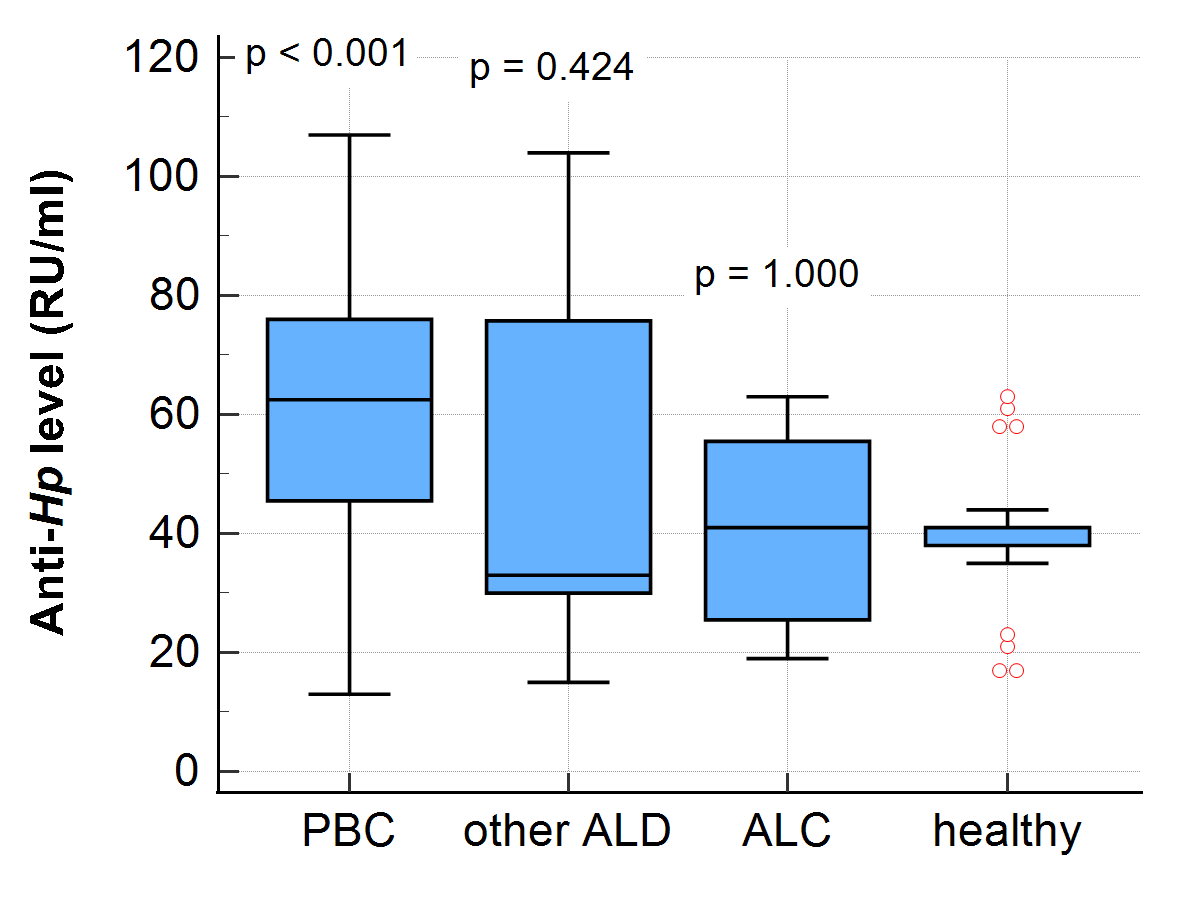


**Supplementary Figure 2.** Mean level of anti-*Hp* antibodies in studied groups


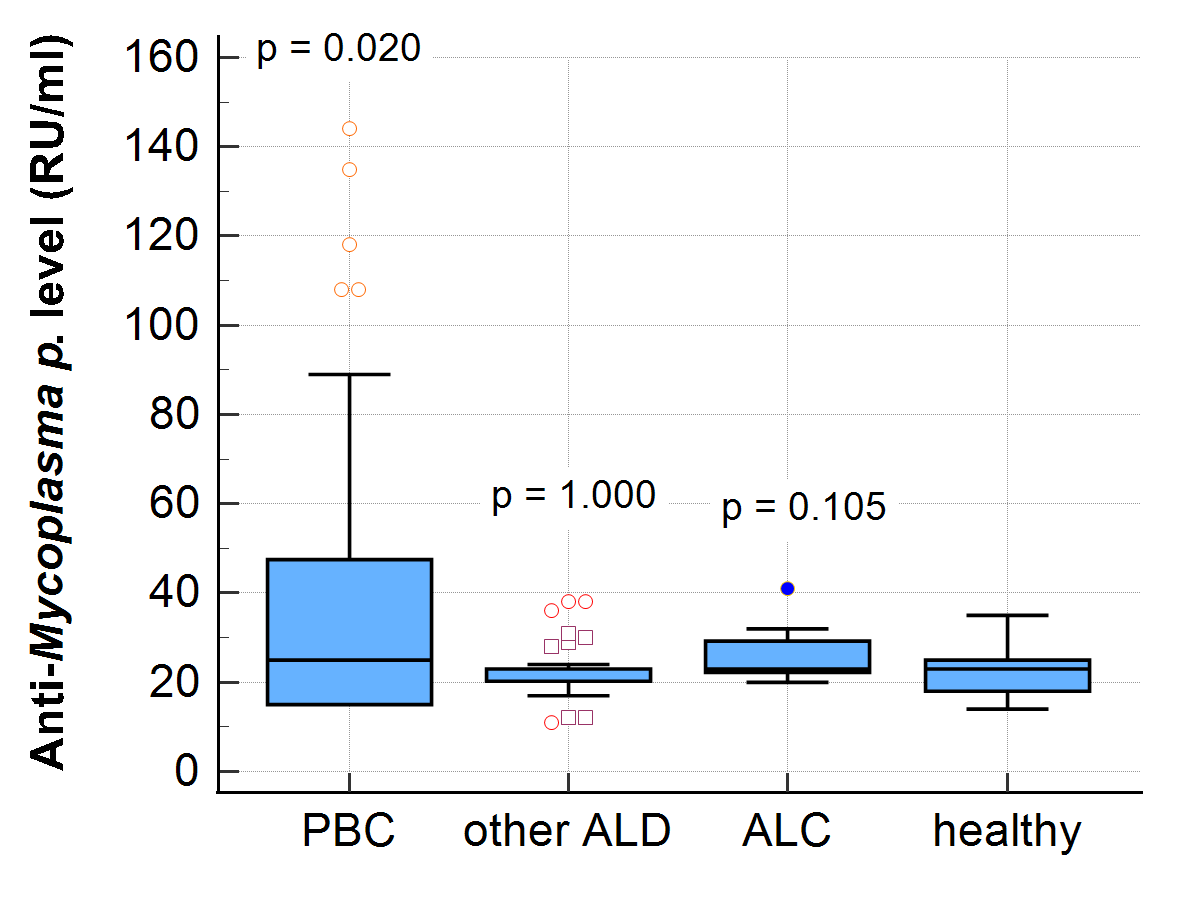


**Supplementary Figure 3.** Mean level of anti-*M. pneumoniae* in studied groups


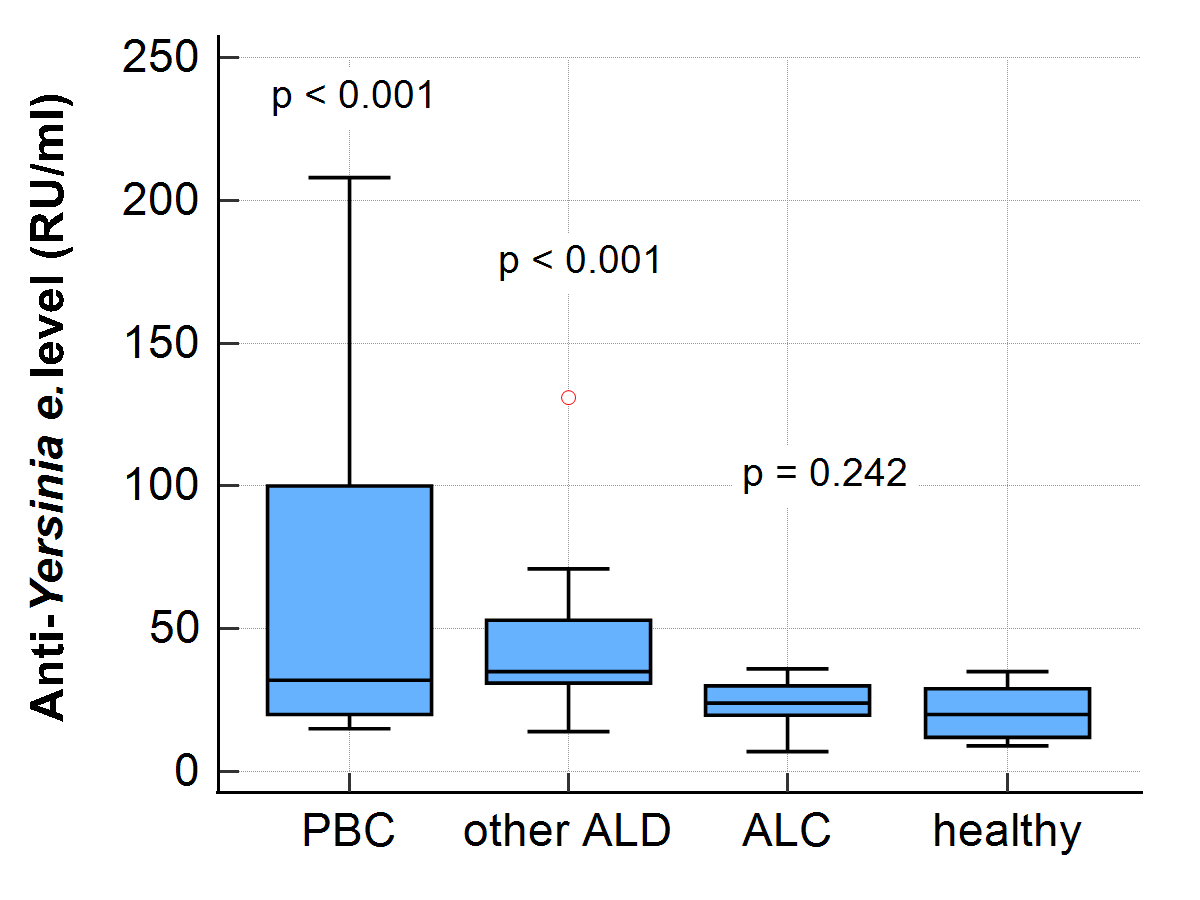


**Supplementary Figure 4.**  Mean level of anti-*Y. enterolitica* antibodies in studied groups


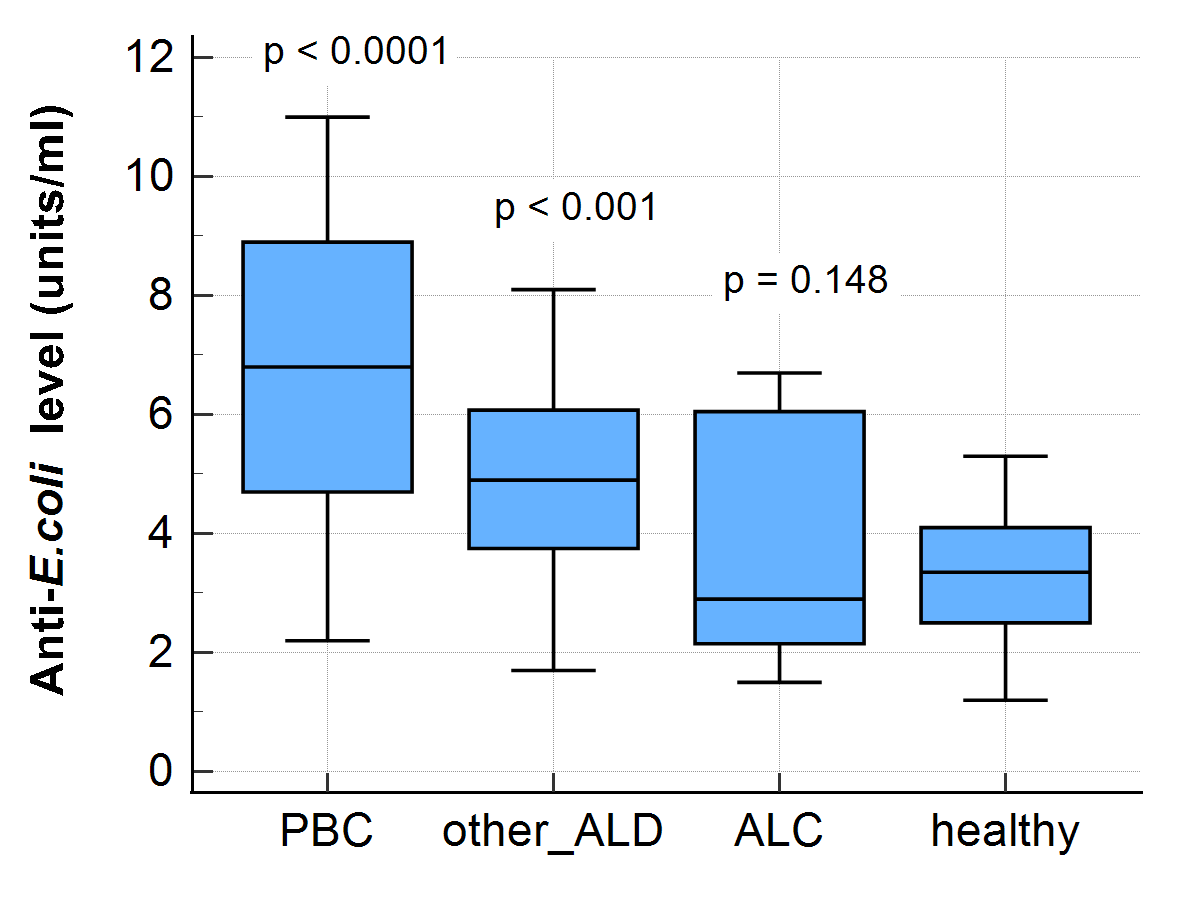


**Supplementary Figure 5.**  Mean level of anti-*E.coli* antibodies in studied groups


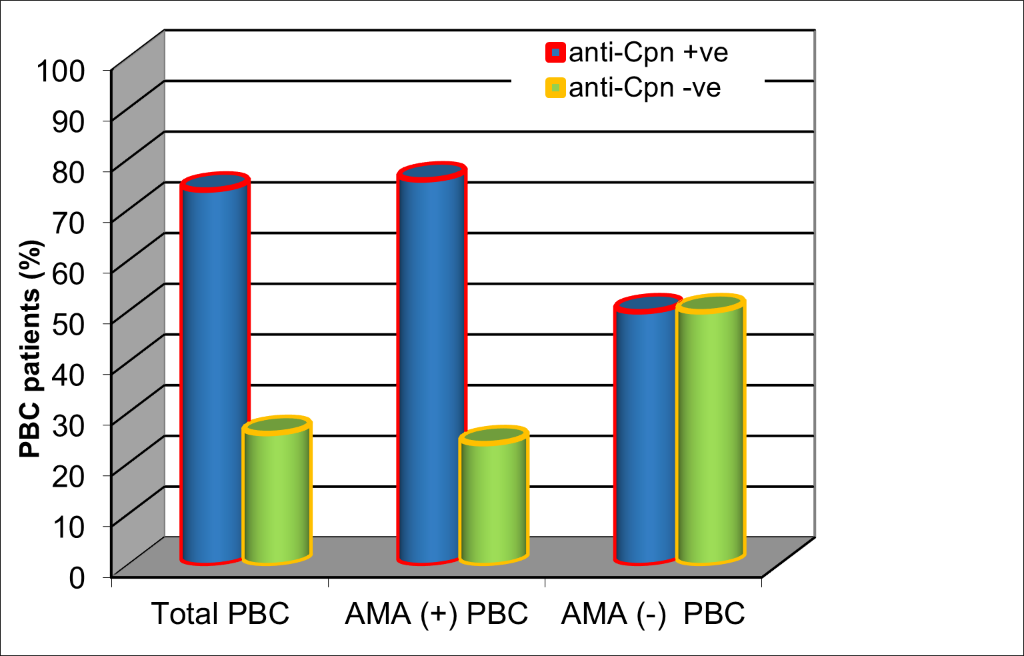


**Supplementary Figure 6.** Seroprevalence of anti-*Chlamydia pneumoniae* antibodies in PBC AMA positive and AMA-negative patients


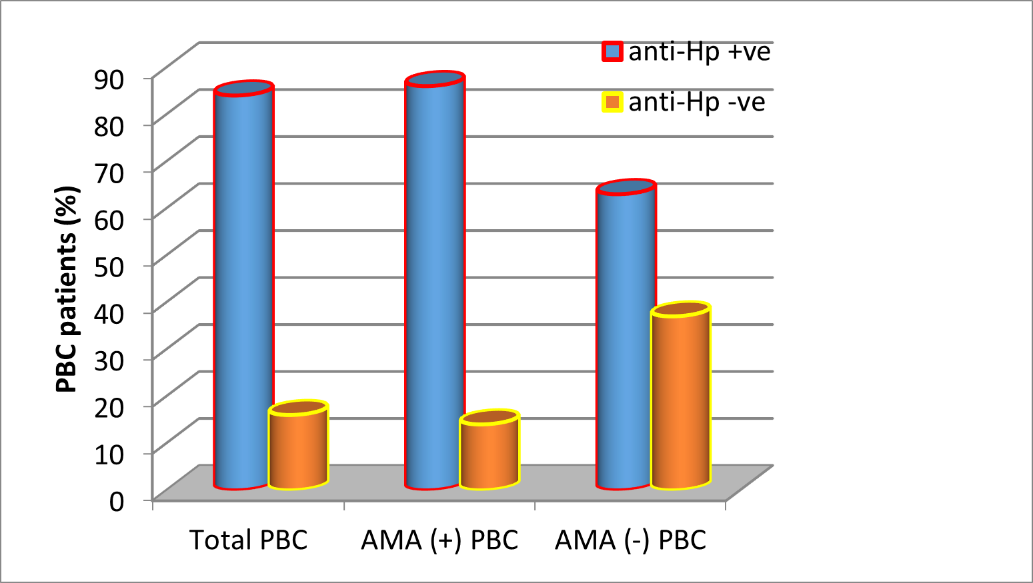


**Supplementary Figure 7.** Seroprevalence of anti-*Helicobacter pylori* antibodies in PBC AMA positive and AMA-negative patients


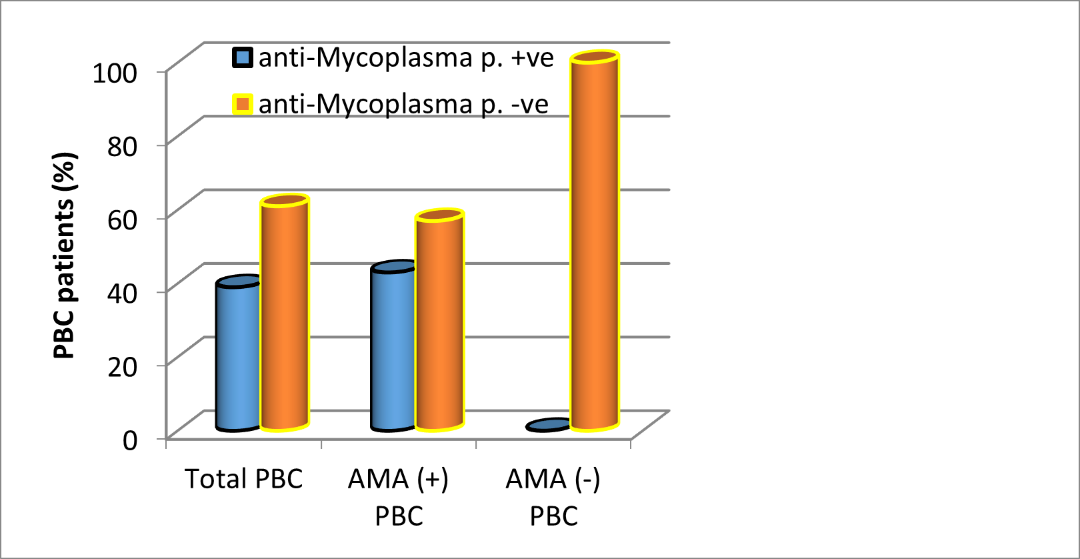


**Supplementary Figure 8.** Seroprevalence of anti-*Mycoplasma pneumoniae* antibodies in PBC AMA positive and AMA-negative patients


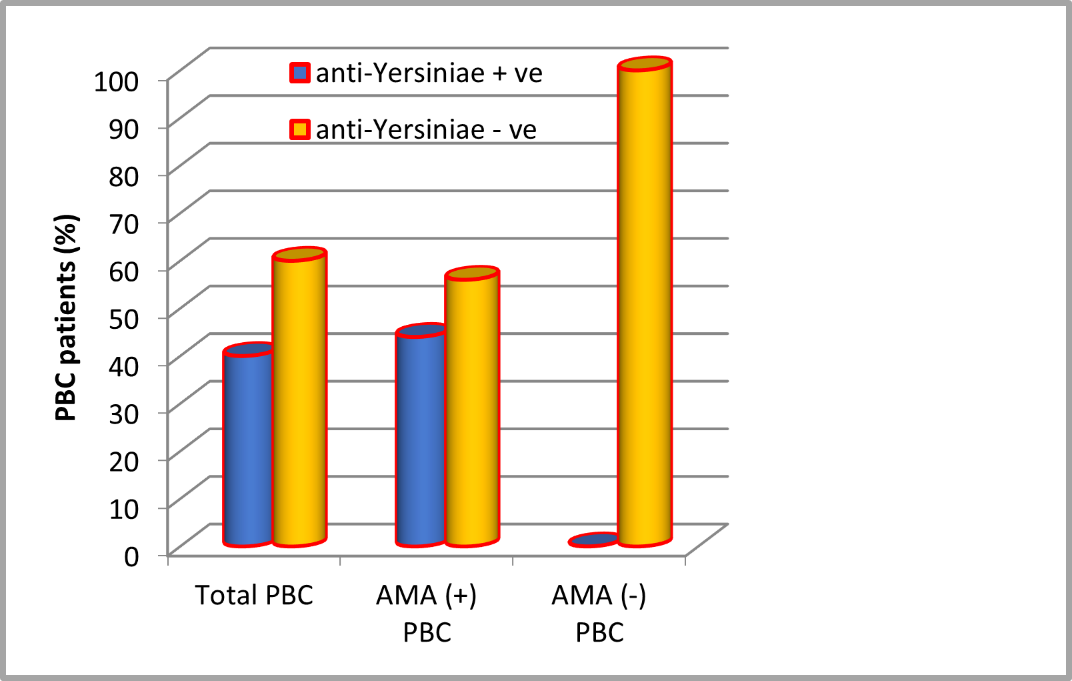


**Supplementary Figure 9.** Seroprevalence of anti- *Y. enterolitica* antibodies in PBC AMA positive and AMA-negative patients
